# Dietary fiber intake and reduced risk of ovarian cancer: a meta-analysis

**DOI:** 10.1186/s12937-018-0407-1

**Published:** 2018-10-30

**Authors:** Bowen Zheng, Hui Shen, Hedong Han, Ting Han, Yonghong Qin

**Affiliations:** 1 Department of Plastic Surgery, Second Hospital of Lanzhou University, Lanzhou, 730030 Gansu Province China; 2 Department of Pharmacognosy, School of Pharmacy, Second Military Medical University, 325 Guohe Road, Shanghai, 200433 China; 3 Department of Health Statistics, Second Military Medical University, Shanghai, 200433 China

**Keywords:** Ovarian cancer, Protective factor, Dietary fiber, Meta-analysis

## Abstract

**Background:**

Epidemiological studies regarding the association between dietary fiber intake and ovarian cancer risk are still inconsistent. We aimed to review the available evidence and conduct a dose-response meta-analysis to investigate the relationship between dietary fiber intake and ovarian cancer risk.

**Methods:**

Relevant studies were identified by searching PubMed, EMBASE, and the Cochrane Library databases before August 2017. Studies that reported relative risk (RR) estimates with 95% confidence intervals (CIs) for the association between dietary fiber intake and risk of ovarian cancer were included. Random-effects models were used to combine the estimated effects extracted from individual study.

**Results:**

Thirteen studies, with a total of 5777 ovarian cancer cases and 142,189 participants, met the inclusion criteria. The pooled multivariable RRs of ovarian cancer for the highest vs. the lowest category of dietary fiber intake was 0.78 (95% CI: 0.70, 0.88) with no evidence of heterogeneity (I^2^ = 4.20%, *P* = 0.40). Our dose-response analysis also showed a significant inverse association between dietary fiber intake and ovarian cancer risk (an increment of 10 g/day; combined RR: 0.88; 95% CI: 0.82, 0.93). There was no evidence for a nonlinear association (*P* for nonlinearity = 0.83).

**Conclusions:**

This meta-analysis suggests a significant inverse dose-response association between dietary fiber intake and ovarian cancer risk. Further studies with prospective design that take account of more potential confounders are warranted to confirm this association.

## Introduction

Ovarian cancer, the second most common female reproductive malignant tumor (240,000 new cases annually) and the leading cause of death in gynecological malignancy (over 150,000 new deaths annually) worldwide [1], has caused heavy public health burden. In spite of recent advances in surgical treatment, prognosis of ovarian cancer remains poor [2]. Thus, it is of prior importance to identify the significant risks and the protective factors associated with the incidence of ovarian cancer.

Epidemiologic studies have suggested that dietary factors play an important role in the etiology of ovarian cancer, including dietary glycemic load [3], fat [4], dietary phytoestrogen [5], fruit and vegetable [6]. Fiber, mainly consumed through diet with cereal, fruit, and vegetable, was reported to be inversely associated with many types of cancers, such as colorectal cancer [7], breast cancer [8], gastric cancer [9] and endometrial cancer [10]. However, reports regarding the association between dietary fiber intake and risk of ovarian cancer were still conflicting. Six case-control studies suggested that dietary fiber intake was inversely related to risk of ovarian cancer [11–16], while others failed to find similar significant association [5, 17–22]. In addition, the effects of dietary fiber intake on ovarian cancer risk seemed to vary with different types and sources of fiber.

Thus, we conducted this meta-analysis with the following purposes: 1) to comprehensively summarize all the available evidence from case-control and cohort studies on the relationship between dietary fiber and risk of ovarian cancer; 2) to examine the discrepancy of ovarian cancer risk according to study design, geographic location and types or sources of fiber; 3) to explore the potential dose-response relationship between dietary fiber intake and risk of ovarian cancer.

## Materials and methods

### Search strategy

The review was registered in PROSPERO-international prospective register of systematic reviews http://www.crd.york.ac.uk/prospero/ (registration number. CRD42016046795).We followed the standard MOOSE [23] and PRISMA criteria [24] to conduct and report this meta-analysis using the databases of PubMed, EMBASE, and the Cochrane library till August 2017. Two investigators (BWZ and HDH) independently identified publications through title and abstract. The eligibility of the publications was further evaluated by full-text assessment. Disagreements between the reviewers were resolved by discussion. We used the following search terms: (diet OR dietary OR fiber OR fibre) AND (ovary OR ovarian) AND (cancer OR neoplasm OR carcinoma OR tumor OR malignancy). Furthermore, the reference lists of retrieved articles were manually scrutinized to identify potential relevant studies.

### Study selection

Eligible studies were included in the meta-analysis if they met the following criteria: 1) the study design was observational (case-control, nested case-control or cohort study); 2) the exposure of interest was dietary fiber intake, and the outcome of interest was ovarian cancer risk; 3) the risk estimates, such as relative risks, odds ratios, or hazard ratios with 95% confidence intervals (CIs) were reported; 4) factors were adjusted for ovarian cancer risk.

### Data extraction

Two reviewers (BWZ, HHD) independently extracted the data from eligible studies using a predefined data extraction form. The following study characteristics were recorded from each study: the first author’s last name, year of publication, study location, study design, No. of participants, No. of cases, dietary assessment, exposure details, contrast (highest vs. lowest), RR (95% CI) (highest vs. lowest), adjustments and New-castle-Ottawa Scale (NOS) score. Validated FFQs meant that FFQs used in the included studies was previously validated. Quality assessment for studies was conducted using the 9-star NOS score [25]. Studies with an NOS score of ≥7 were considered high-quality.

### Statistical analysis

Relative risk was used as the common measure of association across the included studies [26]. The DerSimonian and Laird random-effects model [27, 28], which considered both within-study and between-study variation, was used to pool the estimated effects for the highest vs. the lowest categories of dietary fiber intake.

For the dose-response meta-analysis, we used generalized least-squares regression, which considered the correlation between estimates for different exposure levels, to compute study-specific risk estimates [29, 30]. Meanwhile, to examine a potential nonlinear association, we performed a two-stage random-effect dose-response meta-analysis using restricted cubic splines with three knots at fixed percentiles (10, 50, and 90%) of the distribution [31]. A *P*-value for non-linearity was calculated by testing null hypothesis that the coefficient of the second spline was equal to zero. The midpoint or median dietary fiber intake in each category was used as the assigned dose, and half the width of the adjacent category was used to define the corresponding point for the open categories.

Forest plots were used to assess the RR estimates and corresponding 95% CIs. I^2^ statistics were used to assess statistical heterogeneity among the studies [32]. The subgroup and meta-regression analyses were conducted according to geographic location, type of control subjects, sample size, study design and adjustments for potential confounders. Publication bias was assessed through the Egger’s regression test [33] and funnel plot. We also conducted sensitivity analysis by omitting one study at a time to investigate the influence of a single study on the overall risk estimate.

Stata Version 12.0 software (Stata Corp, College Station, TX) was used for all analyses, and *P*-value < 0.05 was considered to be statistically significant.

## Results

### Study characteristics

**Fig. 1:**
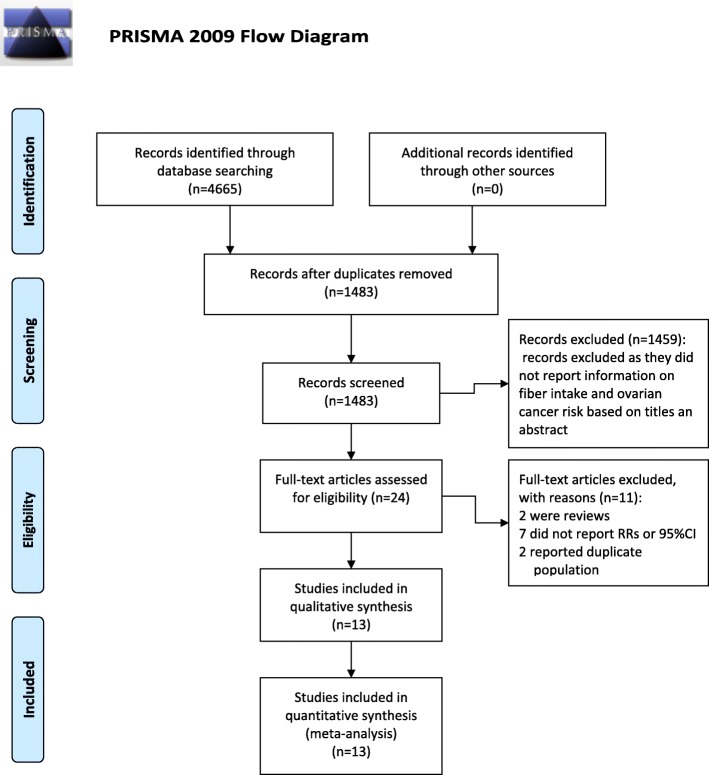
Selection of studies for inclusion in this meta-analysis

Our literature search identified 4665 articles and 4641 were excluded after review of title or abstract (Fig. 1). Twenty-four full-text articles were further reviewed. We excluded 11 studies due to the following reasons: 7 studies did not reported RRs or 95% CI; 2 were review [34, 35]; 2 reported duplicate population [36, 37]. Thus, 13 studies that contained 5,777 ovarian cancer cases and 142,189 participants, published between 1994 and 2015, were included in this meta-analysis. The characteristics of the included studies were summarized in Table 1. Of the 13 studies, 10 were case-control [11–17, 19–21] and 3 were cohort studies [5, 18, 22]. Among these studies, 6 were conducted in USA [12, 15, 17, 18, 21, 22], 2 in Australia [11, 13], 2 in Canada [16, 19], 1 in Italy [14], 1 in Sweden [5] and 1 in Mexico [20]. All the included studies provided RRs that were adjusted for energy intake and most provided RRs that were adjusted for age, oral contraceptive use, menopausal status and parity. All the original studies measured dietary intakes using a food-frequency questionnaire. NOS scores ranged from 6 to 8, and 7 studies were considered high quality.

**Table 1:** Baseline characteristics of all the studies included in the meta-analysis

First Author, Year, Country	Study design	Age (years)	No. of Cases /Participants	Dietary assessment	Exposure details	Contrast (Highest vs. lowest)	RR (95% CI) (Highest vs. lowest)	Adjustments	NOS
Qin,2015, USA	PB-CC	Cases:57.5 Control:54.5	406/1015	Validated 110-item FFQ	Total fiber	≥10.9 vs. ≤6.5 g/d	0.79 (0.53,1.17)	Age, education, region, total energy intake, parity, oral contraceptive use, menopause status, tubal ligation and family history of breast/ovarian cancer, alcohol.	7
Hedelin,2011, Sweden	Cohort	30–49 at entry	163/47140	Validated 80-item FFQ	Total fiber Cereal fiber Vegetable fiber	2.5–10 vs. ≤1.82 g/d 1.5–5.7 vs. ≤0.90 g/d 2.5–10 vs. ≤1.82 g/d	0.82 (0.50,1.35) 1.17 (0.74,1.87) 1.02 (0.63,1.64)	Age, oral contraceptives, age at menarche, parity, hormone replacement therapy, and intake of total energy intake, alcohol, saturated fat, meat, and fish.	8
Nagle,2011, Australia	PB-CC	Cases:57.6 Control:56.3	1366/2780	136-item FFQ	Total fiber	38–77 vs. 10–27 g/d	0.78 (0.62,0.98)	Age, oral contraceptive use, level of post school education, parity, BMI, menopausal status, energy intake.	8
Silvera,2007, USA	Cohort	59.4 ± 7.2	264/49613	Validated 86-item FFQ	Total fiber Soluble fiber Insoluble fiber Cereal fiber Vegetable fiber Fruit fiber	> 24.0 vs. < 15.6 g/d > 7.4 vs. < 4.4 g/d > 5.1 vs. < 2.8 g/d > 5.2 vs. < 2.6 g/d > 10.1 vs. < 5.5 g/d > 5.8 vs. < 2.2 g/d	0.77 (0.52,1.14) 0.79 (0.51,1.23) 0.72 (0.47,1.10) 1.09 (0.71,1.70) 0.94 (0.63,1.40) 0.76 (0.51,1.12)	Age, BMI, alcohol, hormone replacement therapy, use of oral contraceptives, parity, age at Menarche, menopausal status, total energy intake, physical activity, study center, treatment allocation.	8
Pan,2004, Canada	PB-CC	Cases:55.1 Control:55.2	442/2577	Validated 69-item FFQ	Total fiber	Q4 vs. Q1	0.91 (0.66,1.25)	10-year age group, province of residence, education, alcohol consumption, cigarette pack-years, BMI, total caloric intake, recreational Physical activity, number of live births, menstruation years, menopause status.	7
McCann,2003, USA	PB-CC	40–85	124/820	FFQ	Total fiber	> 34 vs. < 19 g/d	0.43 (0.20,0.94)	Age, education, total months menstruating, difficulty becoming pregnant, oral contraceptive use, menopausal status, total energy	6
Zhang,2004, Australia	HB-CC	< 75	254/906	Validated 120-item FFQ	Insoluble fiber	≥15.3 vs. ≤9.7 g/d	0.36 (0.21,0.62)	Age, education, family income, BMI, total energy intake, smoking, alcohol, ovarian cancer in first degree relatives, parity, menopausal status, oral contraceptive use.	6
Salazar-Martinez,2002, Mexico	HB-CC	20–79	84/713	Validated 116-item FFQ	Total fiber	≥24 vs. ≤ 13 g/d	1.15(0.65,2.05)	Age, total energy intake, number of live births, recent changes in weight, physical activity, diabetes.	6
Goodman, 2002, USA	PB-CC	54.8	558/1165	256-item FFQ	Total fiber	≥29.4 vs. ≤ 15.3 g/d	1.01 (0.63,1.63)	Age, ethnicity, study center, education, use of oral contraceptives, parity, tubal ligation, energy intake.	7
Pelucchi,2001, Italy	HB-CC	18–79	1031/3442	Validated 78-item FFQ	Total fiber Insoluble fiber Vegetable fiber Fruit fiber Grain fiber	Q5 vs. Q1 Q5 vs. Q1 Q5 vs. Q1 Q5 vs. Q1 Q5 vs. Q1	0.68 (0.53,0.88) 0.70 (0.54,0.89) 0.59 (0.46,0.77) 0.96 (0.75,1.22) 1.32 (1.03,1.71)	Age, center, education, occupational physical activity, parity, oral contraceptive use, family history of ovarian and/or breast cancer in first-degree relatives, menopausal status, total energy intake	6
McCann,2001, USA,	HB-CC	20–87	496/1921	44-item FFQ	Total fiber	≥30 vs. ≤16 g/d	0.57 (0.38,0.87)	Age, education, region of residence, regularity of menstruation, family history of ovarian cancer, parity, age at menarche, oral contraceptive use, total energy intake	6
Kushi,1999, USA	Cohort	55–69	139/29083	Validated 126-item FFQ	Total fiber	> 23.6 vs. < 16.3 g/d	1.01 (0.61,1.68)	Age, total energy intake, number of live births, age at menopause, family history of ovarian cancer in a first-degree relative, hysterectomy/unilateral oophorectomy status, waist-to-hip ratio, level of physical activity, cigarette smoking, and educational level.	7
Risch,1994, Canada	PB-CC	35–79	450/1014	200-item FFQ	Total fiber Vegetable fiber Fruit fiber Cereal fiber	For 10 g/d increase	0.82 (0.71,0.94) 0.63 (0.49,0.80) 1.03 (1.75,1.43) 0.88 (0.70,1.10)	Age, number of full-term pregnancies, oral contraceptive use, total dally calories, saturated tat/total fat.	6

Abbreviations: *RR* relative risk, *CI* confidence interval, *PB-CC* population-based case-control, *HB-CC* hospital-based case-control, *NOS* Newcastle–Ottawa Quality Assessment Scale, *FFQ* food frequency questionnaire, *BMI* body mass index

### Dietary fiber intake and ovarian cancer risk

**Fig. 2:**
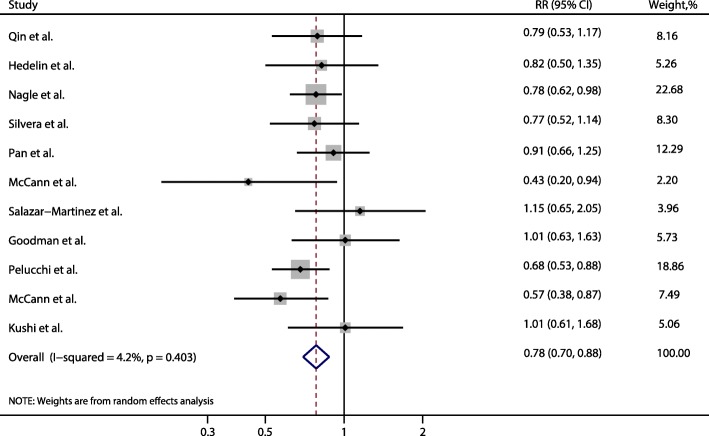
Forest plot of dietary fiber intake and risk of ovarian cancer for highest vs. lowest categories

The multivariable-adjusted RR for each study and the combined RR for the highest vs. the lowest categories of dietary fiber intake were shown in Fig. 2. The pooled RR was 0.78 (95% CI: 0.70, 0.88) with no evidence of heterogeneity across the included studies (I^2^ = 4.20%, *P* = 0.40).

### Dose-response analysis

**Fig. 3:**
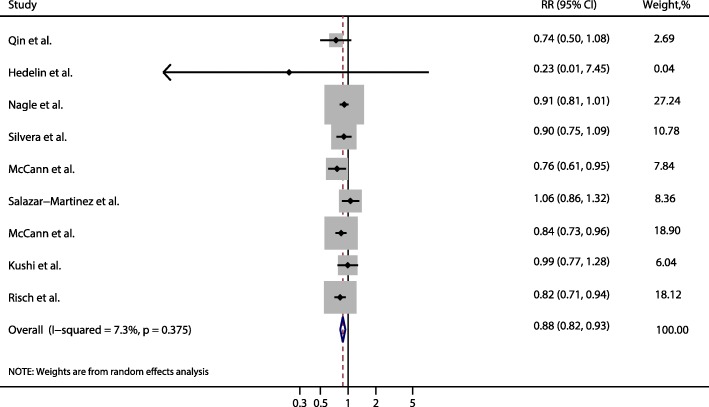
Frost plot of relative risk of ovarian cancer for an increment of 10 g/day dietary fiber intake

Nine studies were included in the dose-response analysis (Fig. 3). The summarized RR for ovarian cancer per 10 g/day increase of dietary fiber intake was 0.88 (95% CI: 0.82, 0.93) without heterogeneity (I^2^ = 7.3%, *P* = 0.38).There was no evidence for a nonlinear association between dietary fiber intake and ovarian cancer risk (*P* for nonlinearity = 0.83).

### Subgroup and meta-regression analyses

The results of subgroup analysis regarding the relationship between dietary fiber intake and risk of ovarian cancer were shown in Table 2. When stratified by study design, the pooled RR was 0.77 (95% CI: 0.66, 0.90) for case-control studies and 0.84 (95% CI: 0.65, 1.10) for cohort studies. In the subgroup analyses by number of cases, the RR was 0.83 (95% CI: 0.65, 1.07) for a sample size < 300 and 0.77 (95% CI: 0.67, 0.87) for a sample size ≥300. In the subgroup analyses by fiber source and fiber type, the RR was 0.80 (95% CI: 0.50, 1.16) for vegetable fiber, 0.90 (0.73, 1.11) for fruit fiber, 1.24 (95% CI: 1.02, 1.51) for cereal fiber, 0.79 (95% CI: 0.51, 1.23) for soluble fiber and 0.60 (95% CI: 0.42, 0.86) for insoluble fiber. The inverse association became insignificant without adjusting for oral contraceptive use (RR: 0.97; 95% CI: 0.76, 1.24) or menopausal status (RR: 0.86; 95% CI: 0.66, 1.11). Meta-regression analysis showed that no variables might account for the heterogeneity across studies (Table 2).

**Table 2:** Subgroup analysis of dietary fiber intake and ovarian cancer risk

Subgroup	Studies, n	RR (95% CI)	I^2^ (%)	*P* ^a^	*P* ^b^
Total	11	0.78 (0.70,0.88)	4.20	0.40	
Study design					
Case-control	8	0.77 (0.66,0.90)	25	0.23	0.17
Cohort	3	0.84 (0.65,1.10)	0	0.70	
Type of control subjects					
Population-based	8	0.82 (0.72,0.94)	0	0.69	0.16
Hospital-based	3	0.72 (0.52,0.99)	48.20	0.15	
Geographic location					
North America	7	0.79 (0.66,0.95)	19.40	0.28	0.87
Others	4	0.77 (0.66,0.89)	0	0.41	
No. of cases					
< 300	5	0.83 (0.65,1.07)	15.40	0.32	0.16
≥ 300	6	0.77 (0.67,0.87)	5.10	0.38	
Study quality					
NOS < 7	4	0.68 (0.50,0.91)	42.90	0.15	0.09
NOS ≥7	7	0.84 (0.73,0.96)	0	0.92	
Validation FFQ					
Yes	7	0.81 (0.70,0.93)	0	0.60	0.59
No	4	0.72 (0.54,0.94)	43.00	0.15	
Fiber type					
Vegetable fiber	3	0.80 (0.55,1.16)	66.90	0.05	
Fruit fiber	2	0.90 (0.73,1.11)	0	0.32	
Cereal fiber	3	1.24 (1.02,1.51)	0	0.73	
Fiber source					
Soluble fiber	1	0.79 (0.51,1.23)	–	–	
Insoluble fiber	3	0.60 (0.42,0.86)	61.00	0.08	
Adjustments					
BMI					
Yes	3	0.81 (0.69,0.96)	0	0.71	
No	8	0.77 (0.64,0.92)	25.40	0.23	
Parity					
Yes	10	0.79 (0.70,0.89)	0	0.52	
No	1	0.43 (0.20,0.93)	–	–	
Oral contraceptive use					
Yes	8	0.74 (0.65,0.84)	0	0.54	
No	3	0.97 (0.76,1.24)	0	0.77	
physical activity					
Yes	6	0.80 (0.70,0.91)	0	0.46	
No	5	0.73 (0.57,0.94)	24.80	0.26	
Menopausal status					
Yes	6	0.76 (0.67,0.86)	0	0.53	
No	5	0.86 (0.66,1.11)	28.70	0.23	

Abbreviations: *RR* relative risk, *CI* confidence interval, *NOS* Newcastle–Ottawa Quality Assessment Scale, *BMI* body mass index, *P*-value^a^, *p* for heterogeneity within each subgroup, *P*-value^b.^, p for heterogeneity between subgroups

### Publication bias and sensitivity analysis

**Fig. 4:**
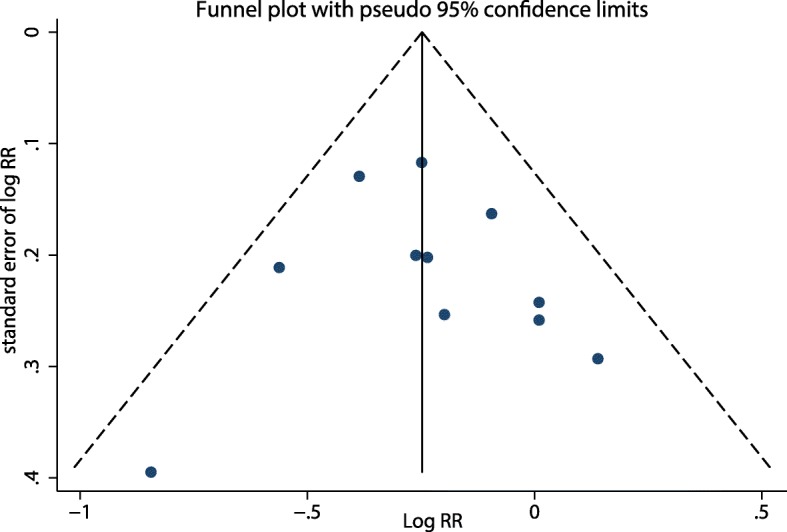
Funnel plot of studies reporting dietary fiber intake and ovarian cancer risk

Funnel plot shapes demonstrated a symmetrical distribution (Fig. 4) and no evidence of publication bias was detected by the Egger’s regression test (*p* = 0.73). Sensitivity analysis shown that none of the studies influenced the combined results substantially, with a range from 0.77 (95% CI: 0.68, 0.87) to 0.81 (95% CI: 0.71, 0.91).

## Discussion

To the best of our knowledge, this is the first meta-analysis to summarize evidence between total dietary fiber intake and different types or sources of dietary fiber intake and risk of ovarian cancer. The meta-analysis of 13 observational studies involving 5,777 ovarian cancer cases supports the hypothesis that a significant inverse association between dietary fiber intake and risk of ovarian cancer. The risk of ovarian cancer was reduced by 22% in the group of highest dietary fiber intake compared with the lowest. Furthermore, a 12% reduction in risk of ovarian cancer was found for per 10 g increase per day. No evidence for a nonlinear association between dietary fiber intake and ovarian cancer was found.

Although no evidence of heterogeneity was found across the included studies, we conducted subgroup analyses to test whether the effect of dietary fiber intake on ovarian cancer risk differed in subpopulations. In the analysis stratified by study design, protective effect of dietary fiber intake was significant in case-control studies but not in cohort studies. There were only three cohort studies with 566 cancer cases, which might not have sufficient power to detect a statistically significant effect. In addition, null association in studies with a relatively small sample size (No. of cases < 300) may also be explained by insufficient statistical power. Additionally, we observed a significant positive association with cereal fiber intake, which probably caused by a high ratio between starch and fiber intake in the Italian population in the study conducted by Pelucchi et al. [14], as the potential promotional action of starch may overwhelm possible protective action of fiber. However, this result should be interpreted carefully and confirmed by further studies because of limited information. The present meta-analysis also indicated that the association was significantly modified by oral contraceptive use and menopausal status, which was corresponding to the previous studies that ovarian cancer risk differed by menopausal status [38] and oral contraceptive use [39].

Several plausible mechanisms have been proposed to explain the hypothesis that dietary fiber intake protects against ovarian cancer. Dietary fiber may decrease circulating estrogen concentrations by changing bacterial macroflora, increasing excretion and consequently lowering serum levels and availability of oestrogens, which reduces the bioavailability of steroid hormones, which in turn is related to the progression of ovarian cancer [8, 40–42]. In addition to the estrogen-related pathway, dietary fiber is believed to reduce glycemic load and improve insulin sensitivity, thus influence insulin-like growth factors which are suggested to be risk factor for ovarian cancer [43, 44].

Our study had several important strengths. This meta-analysis involving 5,777 ovarian cancer cases comprehensively assessed the association between dietary fiber intake and ovarian cancer risk without heterogeneity across studies, enhancing the statistical power to detect a significant association and providing more precise risk estimates. Moreover, most of the included studies were adjusted for important confounders, such as age, energy intake, parity and oral contraceptive use. The sensitivity analysis showed stable and robust results after removal of one study at a time. In addition, outcomes assessment with regards to diagnosis of ovarian cancer in the included studies were histologically confirmed. Finally, the significant inverse dose-response relationship found in this meta-analysis strengthened the association between dietary fiber intake and risk of ovarian cancer.

Potential limitations should be considered in this study. First, we had no access to the individual patient-level data, which would provide a more reliable assessment of relationship between dietary fiber intake and risk of ovarian cancer. Second, the results in our study were mainly based on case-control studies, which may introduce selection bias or recall bias that were inherited in retrospective studies. Third, in observational studies, other factors potentially accounted for the observed association cannot be ruled out. However, potential confounders including energy intake, parity and oral contraceptive use were adjusted in the included studies and the inverse association persisted when analyses were restricted to studies that adjusted for these confounders. Fourth, assessing dietary fiber intake with an FFQ at baseline could have led to overestimation of the range of fiber intake and thus may underestimate the pooled relative risk [45, 46]. Information on the “validated FFQs” was extracted from studies included in this meta-analysis, which meant that the tools used for quantitative dietary measurements were reported with some sort of evaluation in these studies. The evaluation may be tested only in a specific population/setting of interest and for the dietary components of interest but was probably related to smaller bias comparing with the untested FFQs. Moreover, result of subgroup analysis suggested that the inverse association remained significant without application of a validated FFQ. Fifth, the estimates of fiber intakes from different dietary assessment methods and different compositional databases could account for discrepancy among the included studies, so results from this meta-analysis should be interpreted cautiously. Sixth, data in this meta-analysis was mainly from western population, further studies concerning other populations were warranted to generalize this inverse association.

Fiber is mainly consumed through daily diet with cereal, fruit, and vegetable. The mean dietary fiber intake in United States and most European countries is 15 g/day, which is considerably less than the recommended amount (approximately 25–38 g/day) [47, 48]. Considering the public health burden of ovarian cancer, increasing dietary fiber intake in the general population is of importance for ovarian cancer prevention.

## Conclusion

This meta-analysis provides evidence for the hypothesis that a higher intake of dietary fiber is inversely associated with ovarian cancer risk, consistent with a dose-response relationship. This evidence is largely limited to case-control studies. Further studies with prospective design that are adequately adjusted for potential confounders and clarified types or sources of fiber are needed to confirm our findings.

## Abbreviations

BMI: body mass index
CI: confidence interval
FFQ: food frequency questionnaire
HB-CC: hospital-based case-control
NOS: Newcastle–Ottawa Quality Assessment Scale
PB-CC: population-based case-control
RR: relative risk
